# The effect of mild decrement in plasma volume simulating short-duration spaceflight on intracranial pressure

**DOI:** 10.1038/s41526-018-0053-0

**Published:** 2018-10-02

**Authors:** Takuya Kurazumi, Yojiro Ogawa, Hiroshi Morisaki, Ken-ichi Iwasaki

**Affiliations:** 1 Department of Social Medicine, Division of Hygiene, Nihon University School of Medicine, Tokyo, Japan; 2 Department of Anesthesiology, Keio University School of Medicine, Tokyo, Japan

## Abstract

Short-duration spaceflight induces an approximately 10% reduction in plasma volume, which leads to mild volume depletion. In a previous study, we found that mild volume depletion improved dynamic cerebral autoregulation. However, the effect of mild volume depletion on intracranial pressure (ICP) remains unknown. Therefore, we estimated ICP noninvasively (nICP), and calculated two indices relating to ICP, the cerebral artery compliance and the cerebral blood flow pulsatility index (PI), to examine whether ICP would decrease due to a mild decrement in plasma volume. In our previous experiment, fourteen subjects were administered 0.2 mg/kg of furosemide in a supine position to simulate an approximately 10% reduction in plasma volume induced by short-duration spaceflight. We re-analyzed the cerebral blood flow velocity waveform from the middle cerebral artery obtained by transcranial Doppler and the arterial blood pressure waveform at the radial artery obtained by tonometry to estimate nICP and to calculate cerebral artery compliance and PI using mathematical analysis based on an intracranial hydraulic model. All indices were compared between before and after furosemide administration. There were no significant changes in nICP and cerebral artery compliance. However, PI decreased significantly from before to after furosemide administration (0.78 ± 0.10 to 0.74 ± 0.09, *p* = 0.009). Decreases in ICP were not observed during the 10% reduction in plasma volume. Although cerebral artery compliance did not change, PI decreased significantly. These findings suggest that the impedance of distal cerebral arteries would be reduced in response to mild decreases in plasma volume induced by short-duration spaceflight.

## Vasculature: Spaceflight-induced fluid loss may alter cerebral arteries

Short-duration spaceflight may affect the ability of arteries on the brain to control resistance to pulsatile circulating blood. Kenichi Iwasaki from Nihon University School of Medicine in Tokyo, Japan, and colleagues administered a diuretic medication to 14 people to induce an approximate 10% reduction in blood plasma volume, as experienced by astronauts in outer space. The researchers found that the pulsations in blood velocity decreased significantly inside people’s cerebral arteries after intravenous injection of the blood volume–reducing drug, a sign of changed vascular resistance to blood pressure pulsations. They saw no such differences, however, in either a noninvasive proxy measure of pressure inside the skull, or in the propensity of arteries on the brain to expand and contract with changes in pressure. The authors suggest that microgravity may have similar effects on cerebral arteries.

## Introduction

The distribution of body fluid is affected by gravity, which is altered in microgravity.^1^ Short-duration spaceflight induces an approximately 10% reduction in plasma volume, which is considered to be mild intravascular fluid deprivation.^2,3^ Our previous study demonstrated that administration of furosemide (0.2 mg/kg) produced a 10% reduction in plasma volume.^4^ Our previous study also showed decreases in the magnitude of the transfer from arterial blood pressure (ABP) oscillations to cerebral blood flow velocity (CBFv) fluctuations, which suggested an improved cerebral autoregulation of the enhanced suppression capability from ABP to cerebral blood flow (CBF).^4^ This finding was considered to be partially associated with the enhanced cerebral autoregulation that occurs during and after short-duration spaceflight.^5^ Thus, mild volume depletion alters cerebrovascular regulation; however, the effect on intracranial pressure (ICP) remains unknown.

Czosnyka et al.^6^ established an intracranial hydraulic model, comprising pathways of CBF and cerebrospinal fluid (CSF). Monitoring the ABP waveform and CBFv waveform using transcranial Doppler in high resolution could be applied to the model-based analysis. Cerebral artery compliance and the CBF pulsatility index (PI) reportedly reflect the changes in the intracranial hydraulics and cerebral circulation system.^7^ The PI has been applied for assessment of ICP, distal cerebrovascular resistance, or cerebral perfusion pressure.^7^ Recently, estimation of noninvasive ICP (nICP) based on system analysis reflecting intracranial physiology has been developed using precise ABP and CBFv waveforms.^8^

To date, there have been no reports on the effect of mild volume depletion on ICP. Therefore, we estimated nICP, as well as calculated cerebral artery compliance and PI using our previous research data^4^ to examine whether ICP would be decreased by a reduction in plasma volume.

## Results

The 6-min time average of nICP (nICP_mean) did not change significantly from before furosemide administration to after furosemide administration (Table 1, *t* = 0.4639, d*f* = 13, *p* = 0.650). There was no significant difference in the standard deviation of nICP during the 6-min period (nICP_SD) between before and after furosemide administration (Table 1, *t* = 0.5468, d*f* = 13, *p* = 0.593). Although cerebral artery compliance did not change significantly (Table 1, *t* = 1.4196, d*f* = 13, *p* = 0.179), PI decreased significantly (Table 1, *t* = 3.0188, d*f* = 13, *p* = 0.009).

**Table 1:** The sample average and standard deviation of three indices relating to intracranial pressure before and after administration of furosemide

		Before administration	After administration	*p* value
**nICP_mean**	(mmHg)	10.2 ± 3.0	10.0 ± 2.9	0.650
**nICP_SD**	(mmHg)	1.31 ± 0.22	1.29 ± 0.22	0.593
**Cerebral artery compliance**	(cm^3^/mmHg)	0.16 ± 0.04	0.15 ± 0.04	0.179
**PI**		0.78 ± 0.10	0.74 ± 0.09*	0.009

nICP_mean, 6-min time average of noninvasive intracranial pressure estimation using mathematical model by plugin software “nICP Plugin”. nICP_SD, standard deviation of noninvasive intracranial pressure during 6-min period. PI, pulsatility index of cerebral blood flow.

* *p* < 0.05

## Discussion

The major finding of the present re-analysis is that a mild decrement in plasma volume induced by administration of 0.2 mg/kg of furosemide did not change nICP or cerebral artery compliance, but decreased PI significantly. Contrary to our expectation, ICP did not decrease due to a mild decrement in plasma volume induced to simulate the effect of short-duration spaceflight.

**Fig. 1:**
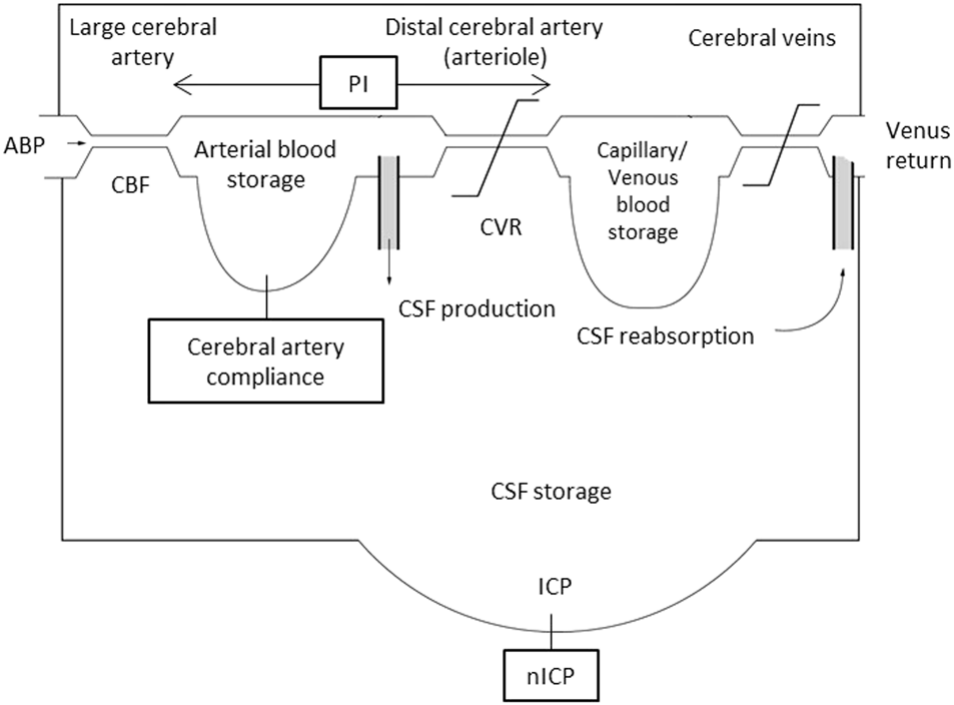
Intracranial hydraulic model, comprising pathways of cerebral blood flow and cerebrospinal fluid. Adapted from Czosnyka et al.6 (License Number 4399630137220). ABP arterial blood pressure, CBF cerebral blood flow, CVR cerebrovascular resistance, CSF cerebrospinal fluid, ICP intracranial pressure, nICP noninvasive intracranial pressure, PI pulsatility index

The intracranial hydraulic model is shown in Fig. 1, which describes the cerebral components corresponding to the mathematical equation adapted from Czosnyka et al.^6^ There are two main types of intracranial compliance; one is related to the cerebrovascular bed and the other to the CSF storage.^7^ The cerebral artery compliance represents the change in arterial blood volume in response to a change in ABP, and the CSF storage compliance refers to changes in the volume of the CSF storage in response to changes in ICP.^7^ In this re-analysis study, the standard deviation of 6-min nICP fluctuations as well as the mean nICP were considered to reflect total intracranial hydraulic pressure including the CSF storage compliance. A change in the standard deviation of the 6-min nICP fluctuations between before and after furosemide administration was not observed. Moreover, a change in the mean nICP between before and after furosemide administration was also not observed. These two results in the present study indicate that the total intracranial hydraulic pressure was not associated with a reduction in plasma volume. Since cerebral artery compliance represents the change in arterial blood volume in response to a change in ABP, which is considered to reflect arterial blood storage, the unchanged cerebral artery compliance suggests preserved arterial blood storage during the reduction in plasma volume. The preserved arterial blood storage is considered to be consistent with our previous result that CBFv did not differ between before and after furosemide administration.^4^ We investigated cerebral artery compliance, but did not measure the change in CSF compliance because of the limitations of our noninvasive method. However, estimation of continuous cerebral artery compliance enabled a detailed investigation of the main parameters for mean ICP and the shape of the ICP pulse waveform.^9^ Although there are no data on CSF compliance in the present study, the results showing that neither nICP nor cerebral artery compliance were associated with a reduction in plasma volume suggest unchanged ICP in mild volume depletion. In contrast, PI reflecting the impedance of pulsate flow significantly decreased in mild volume depletion. The unchanged cerebral artery compliance and the decreased PI together suggest reductions in cerebrovascular resistance at the distal level considering the intracranial hydraulic model (Fig. 1). Administration of 0.2 mg/kg of furosemide slightly increased hematocrit in these subjects (Table 2), which would induce a slight increase in blood viscosity. The reduction in resistance of distal cerebral arteries is a reasonable response to a slight increase in blood viscosity based on the Hagen–Poiseuille equation (1) when considering laminar flow across a constant pressure gradient^10^:

1 $${\mathrm{CBF}} = \frac{{{\mathrm{\Delta }}P\,\pi \,R^4}}{{8\,\eta \,l}},$$

where Δ*P* represents the cerebral perfusion pressure, *R* represents the radius of the vessel, η represents blood viscosity, *l* represents the length of the vessel and π is a constant 3.14. Thus, the radius of the distal cerebral artery increased to maintain CBF against the increased blood viscosity. This interpretation is consistent with the decreases in PI. Although we could not reveal whether the present levels of decreases in PI were clinically important phenomena, the decreased PI in the present study is considered to be a physiological response in cerebral arteries. These cerebral artery responses including the reduction in resistance of distal cerebral arteries might partially improve cerebral autoregulation in mild volume depletion.^4^ The change in PI has been used to assess cerebrovascular resistance because PI shows good correlation with change in ICP in the time domain recording among the neurosurgical patients.^11^ On the other hand, it remains controversial whether PI always reflects changes in ICP in any physiological situation.^12^ We considered that the change in PI would be a predictor for cerebrovascular regulation rather than a reflection of ICP.

**Table 2:** Hemodynamic and circulatory blood data before and after administration of furosemide reported from our previous study^4^

		Before administration	After administration	*p* value
MAP	(mmHg)	81.5 ± 9.6	82.5 ± 10.0	0.429
MCBFv	(cm/s)	73.5 ± 11.4	71.7 ± 10.0	0.456
Hematocrit	(%)	40.2 ± 3.6	43.7 ± 2.4*	<0.001
Hemoglobin	(g/dL)	14.3 ± 1.1	15.0 ± 0.8*	0.01
Change in plasma volume	(%)	—	−9.9 ± 4.6*	0.001
Central venous pressure	(mmHg)	5.7 ± 0.7	4.5 ± 0.7*	0.009

*MAP* mean arterial blood pressure. MCBFv, mean cerebral blood flow velocity.

* *p* < 0.05

Although administration of furosemide has also been used empirically in the management of ICP to reduce CSF volume during neurosurgery, it is still unclear whether furosemide decreases ICP or CSF volume. Administration of furosemide did not show any reductions in CSF volume in an animal study.^13^ In the present study, the total intracranial hydraulic pressure as shown by nICP was not changed from before to after furosemide administration. However, central venous pressure decreased slightly, which potentially suggests a decreased venous volume. Therefore, the intracranial hydraulics including CSF volume are considered to be unchanged or possibly increased in mild volume depletion induced by administration of 0.2 mg/kg of furosemide, in accordance with the Monro–Kellie doctrine, which states that the sum of the volumes of the brain, CSF, and blood is constant.^14^ This finding may apply to the intracranial condition during short-duration spaceflight, and supports the results from another study showing that changes in ventricular CSF volume from short-duration spaceflight were smaller than those from long-duration spaceflight.^15^ Thus, the changes in ICP or CSF volume may differ between short and long durations in microgravity. Further investigation into the effect of long-term exposure to microgravity on ICP is required to better understand space flight-associated neuro-ocular syndrome. It remains unclear whether optic disc edema represents true increased ICP.^16^

A limitation of the present re-analysis is the accuracy of the noninvasive ICP estimation. Although the nICP values may be less accurate than the absolute values obtained invasively, the reliability of the relative changes was confirmed among neurosurgical patients with a low range of ICP values.^17^ Unfortunately, there is a paucity of evidence regarding estimates of nICP in healthy individuals. However, the reliability of the transmission ABP-ICP in healthy subjects has been confirmed by the high coherent relationship between ABP and CBFv.^18^ Moreover, we did not attempt to show that the intracranial hydraulic model relates to nICP or the method for estimating nICP. Therefore, the interaction between the intracranial hydraulic model and the method for estimating nICP is another limitation to the interpretation of our re-analysis findings.

Furthermore, we should take into account the limitations related to the retrospective statistical analysis in the ground-based study. First, we detected the decrease in PI in subjects in a supine position, thereby eliminating hydrostatic pressure gradients; however, the levels of distribution of body fluid in the present study might not match completely with the high cephalad fluid shift in microgravity. Therefore, the combined effects of a reduction in plasma volume and high cephalad fluid shift on ICP remains unknown. Second, our healthy subjects were younger (21 ± 2 years old) than recent astronauts (47 ± 1 years old in short-duration spaceflight, 48 ± 1 years old in long-duration spaceflight).^15^ Further study matching with these factors will be desirable to apply the findings from a ground-based study to those from the early phase of short-duration spaceflight. Finally, the estimation of PI would also depend on the sampling rate, waveform shape or calculation window.

In conclusion, nICP and cerebral artery compliance did not change due to a 10% reduction in plasma volume induced by administration of 0.2 mg/kg of furosemide to simulate short-duration spaceflight, but PI decreased significantly. Decrease in ICP was not seen in mild volume depletion. Together, the lack of change in cerebral artery compliance and the decrease in PI suggest reduced impedance of distal cerebral arteries. Thus, these cerebral artery responses may occur with a mild decrement in plasma volume induced by short-duration spaceflight.

## Methods

### Study design

The aim of the present study was to re-analyze the data of our previous research on mild volume depletion and dynamic cerebral autoregulation^4^ to investigate the effect of mild volume depletion during short-duration spaceflight on ICP with a ground-based study. All subjects provided written informed consent. The Ethics Committee of Nihon University School of Medicine approved the study protocol (approved December 14, 2004, No. 5).

**Fig. 2:**
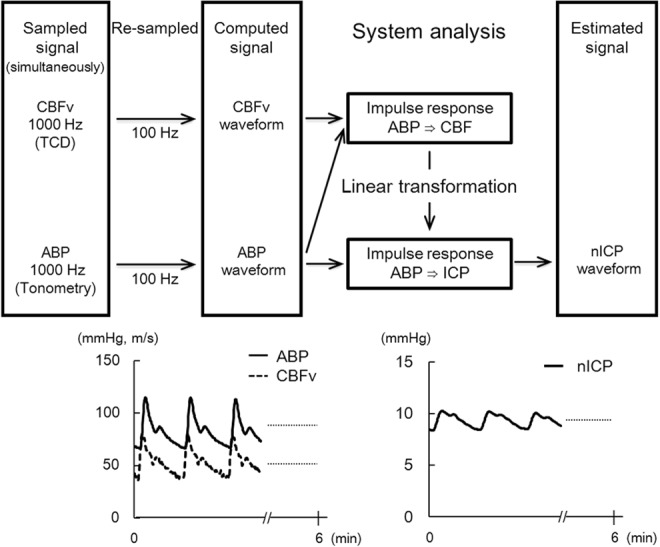
The computational model to estimate intracranial pressure non-invasively with examples of representative waveforms. ABP arterial blood pressure, CBF cerebral blood flow, CBFv cerebral blood flow velocity, TCD transcranial Doppler, ICP intracranial pressure, nICP noninvasive intracranial pressure

The details of the procedures are described in a previous report.^4^ Briefly, 14 male volunteers (age 21 ± 2 years; height 173 ± 5 cm; weight 65 ± 8 kg; means ± standard deviations) lay supine for 30 min of rest to record data before furosemide administration. Then, 0.2 mg/kg of furosemide was administered to produce an approximately 10% reduction in plasma volume to simulate microgravity effects. Subjects walked to the lavatory and urinated when they experienced uresiesthesia. The data after furosemide administration were measured in the supine position after the subjects had used the lavatory and an additional 15 min of quiet rest. The mean duration between collection of data before and after furosemide administration was 44 ± 8 min. The continuous ABP waveform at the radial artery was obtained by tonometry using a noninvasive ABP monitor, and was calibrated against intermittent blood pressure measured using the oscillometric method with a cuff sphygmomanometer placed over the brachial artery (Jentow 7700; Colin, Aichi, Japan). The continuous CBFv waveform from the middle cerebral artery was obtained by transcranial Doppler ultrasonography (WAKI; Atys Medical, St. Genislaval, France) with a 2-MHz probe placed over the temporal window and fixed at a constant angle with a probe holder customized to fit individual right facial bone structures and ears. Data for 6 min of both the ABP waveform and CBFv waveform were recorded simultaneously at a sampling frequency of 1000 Hz, and the data were resampled at 100 Hz using Notocord-Hem 3.3 software (Notocord, Paris, France) (Fig. 2).

Blood collection to determine the hematocrit and hemoglobin concentration, as well as administration of furosemide were performed in seven subjects using a 22-gauge catheter inserted into a forearm vein. The change in plasma volume was calculated using the Dill method,^19^ as described in a previous study. Measurement of central venous pressure and administration of furosemide were performed in another seven subjects via a central catheter (First PICC catheter, 18 GA, 1.35 mm 365 cm, Becton Dickinson, Franklin Lakes, NJ, USA) inserted into an antecubital vein to the level of the superior vena cava. The mean ABP, mean CBFv, and mean values for hematocrit, hemoglobin concentration, percent change in plasma volume and central venous pressure in the previous study^4^ are shown in Table 2.

### Data analysis

The mathematical model for the nICP estimation is based on the system analysis reflecting the physiology of the intracranial components, in which the rules for signal transformation from ABP to ICP are controlled by the relationship between ABP and CBF.^8,20^ The nICP waveform was estimated from ABP and CBFv waveforms using the signal calculator of the “nICP Plugin” software (Klinkum Chemnitz gGmbH, Chemnitz, Germany) (Fig. 2). The reliability of the transmission ABP-ICP from our sampling data has been confirmed, as indicated by a high coherent relationship between ABP and CBFv.^18^ The time average of nICP (nICP_mean) was calculated using the full length of the nICP waveform during the 6-min period. The standard deviation of nICP during each 6-min period (nICP_SD) for each subject was calculated to present the 6-min variation in nICP.

Cerebral artery compliance reflects the change in cerebral blood volume (CBV) in response to the change in ABP. The change in CBV is derived from the time-series CBFv and moving average of CBFv as follows (2):

2 $${\mathrm{\Delta }}{\mathrm{CBV}}(t) = \mathop {\int}\limits_0^t {({\mathrm{CBFv}}(s) - {\mathrm{CBFv}}_{{\mathrm{moving}}\,{\mathrm{average}}}(s))ds}.$$

Then, cerebral artery compliance is calculated as follows (3):

3 $$Ca = \frac{{{\mathrm{AMP}}_{{\mathrm{CBV}}}}}{{{\mathrm{AMP}}_{{\mathrm{ABP}}}}}\left( {\frac{{{\mathrm{cm}}^3}}{{{\mathrm{mmHg}}}}} \right),$$

where AMP represents the amplitude.

PI is a descriptor of the CBFv amplitude, as shown by the following equation (4):

4 $${\mathrm{PI}} = \frac{{{\mathrm{CBFv}}_{{\mathrm{systolic}}} - {\mathrm{CBFv}}_{{\mathrm{diastolic}}}}}{{{\mathrm{CBFv}}_{{\mathrm{mean}}}}}.$$

Cerebral artery compliance and PI were calculated every 10-s window, and the values of 36 windows were averaged, in accordance with the model-based cerebrovascular dynamics described by Varsos et al.^7^ using ICM+ version 8.1 (Cambridge Enterprise: http://www.neurosurg.cam.ac.uk/icmplus/, Cambridge, United Kingdom).

### Statistical analysis

Data are presented as the mean and standard deviation. The normal distribution of data was confirmed using the Kolmogorov–Smirnov test. Variables were compared using a paired *t*-test (before vs. after furosemide administration). Values of *p* < 0.05 were considered significant. All statistical analyses were performed using R version 3.1.2, 64-bit (The R Foundation for Statistical Computing, Vienna, Austria) with the EZR graphical interface (Jichi Medical University Saitama Medical Center, Saitama, Japan).^21^

## Data Availability

The data that support the findings of this study are not publicly available due to containing personal details of subjects, but are available from the corresponding author upon reasonable request.
